# Effect of CD14/TLR4 antagonist on GnRH/LH secretion in ewe during central inflammation induced by intracerebroventricular administration of LPS

**DOI:** 10.1186/s40104-018-0267-8

**Published:** 2018-07-16

**Authors:** Karolina Haziak, Andrzej Przemysław Herman, Karolina Wojtulewicz, Bartosz Pawlina, Kamila Paczesna, Joanna Bochenek, Dorota Tomaszewska-Zaremba

**Affiliations:** The Kielanowski Institute of Animal Physiology and Nutrition, Polish Academy of Sciences, 05-110 Jabłonna, Poland

**Keywords:** Ewe, GnRH, LH, LPS, TLR4 antagonist

## Abstract

**Background:**

Immune stress induced by lipopolysaccharide (LPS) influences the gonadotropin-releasing hormone (GnRH)/luteinizing hormone (LH) secretion. Presence of LPS interacting Toll-like receptor (TLR) 4 in the hypothalamus may enable the direct action of LPS on the GnRH/LH secretion. So, the aim of the study was to investigate the influence of intracerebroventricular (icv) injection of TLR4 antagonist on GnRH/LH secretion in anestrous ewes during LPS-induced central inflammation. Animals were divided into three groups icv-treated with: Ringer-Locke solution, LPS and TLR4 antagonist followed by LPS.

**Results:**

It was demonstrated that TLR4 antagonist reduced LPS-dependent suppression of *GnRH* gene expression in the preoptic area and in the medial basal hypothalamus, and suppression of receptor for *GnRH* gene expression in the anterior pituitary gland. It was also shown that TLR4 antagonist reduced suppression of LH release caused by icv injection of LPS. Central administration of LPS stimulated *TLR4* gene expression in the medial basal hypothalamus.

**Conclusions:**

It was indicated that blockade of TLR4 prevents the inhibitory effect of centrally acting LPS on the GnRH/LH secretion. This suggests that some negative effects of bacterial infection on the hypothalamic-pituitary-gonadal axis activity at the hypothalamic level may be caused by central action of LPS acting through TLR4.

## Background

It is stated that bacterial and viral infections affect female reproductive system, causing disturbances in the secretory activity of the hypothalamic-pituitary-gonadal (HPG) axis, consequently leading to many reproductive dysfunctions, ranging from ovulation cycle disorders to even complete infertility [1, 2]. It is considered that the suppression of the HPG axis activity during bacterial infection may result from direct and indirect effect of bacterial endotoxin – lipopolysaccharide (LPS) [3].

Bacterial endotoxin is a principal pathogenic component of outer membrane Gram-negative bacterial cell walls and it is recognized by immune system cells as a signal of danger. LPS is released from the surface of replicating or dying bacteria into the circulation and plays a key role in the pathophysiology of infectious diseases [4]. LPS induces cellular responses through Toll-like receptor (TLR) 4, that plays a crucial role in the activation of innate immunity and pathogen recognition [5, 6]. Animal cells are stimulated by LPS through signaling cascades with several specific proteins like cluster of differentiation (CD) 14 protein, myeloid differentiation factor 2 (MD-2) and LPS-binding protein (LBP), a necessary component of TLR4 [7, 8]. Endotoxin forms a complex with LBP, and then, this complex binds to CD14, which plays a pivotal role in TLR4 receptor activation [9, 10]. Thus, in collaboration with these proteins, TLR4 is activated and can conduct its inflammatory signal through the system of intracellular pathways, which ultimately leads to activation of some transcription factors or induces apoptosis [11, 12].

Due to its immunogenic activity, bacterial endotoxin is commonly used in experimental models of animal and human infections. Peripheral administration of LPS triggers a cascade of cytokines and prostaglandins to induce a wide variety of pathophysiological responses, activation of the hypothalamic-pituitary-adrenal (HPA) axis [13] and inhibition of the HPG axis [14]. It is proven, that bacterial endotoxin exerts an inhibitory effect on the reproductive system at the level of hypothalamus by altering the gonadotropin-releasing hormone (GnRH) secretion [1] and at the level of anterior pituitary gland (AP) [15] by inhibiting both, the pulsatile release of luteinizing hormone (LH) and the LH surge [16, 17]. It is postulated that the immune and neuroendocrine systems may communicate with each other through the action of the pro-inflammatory mediators – cytokines, molecules synthesized by the cells belonging to these two systems [13, 18, 19]. It was shown that LPS given intra-arterially increased plasma concentration of pro-inflammatory cytokines including interleukin (IL)-1β, IL-6 and tumor necrosis factor (TNF) α [20]. Moreover, it was reported that peripherally administered LPS also raised the level of the above-mentioned pro-inflammatory cytokines present in the mammalian cerebrospinal fluid (CSF) during inflammation [21, 22]. The origin of these pro-inflammatory cytokines in the central nervous system (CNS) seems to be differentiated: the peripheral cytokines can cross the blood-brain barrier (BBB) through fenestrated brain capillaries in the choroid plexus (CP), the *organum vasculosum* of the *lamina terminalis*, the median eminence (ME), the subfornical organ and the area postrema [23]. In the recent study it is suggested that CP may play a significant role in the transition of the inflammatory signal into the brain parenchyma. It was found that the cells of the CP express all *TLRs* genes except *TLR8*. So it can be suggested that this structure has a potential to sense the presence of many bacterial and viral components [24]. Moreover, it was reported that in response to peripheral bacterial endotoxin stimulation, pro-inflammatory cytokines could be synthesized by macrophage-like cells in the circumventricular organs and the CP [25]. The cytokines may also be transported into the brain via the BBB by saturated, self-inhibitable transport mechanism [23]. However, significant sources of central pro-inflammatory cytokines may generate their own synthesis within the brain tissue including hypothalamus [26–28]. Centrally acting pro-inflammatory cytokines may disturb the HPG axis activity by affecting the secretion of GnRH in the hypothalamus. In the previous studies it was shown that IL-1β, acting at the hypothalamic level, is a potent negative regulator of the HPG axis activity both in rat [29] and sheep [30]. Furthermore, the important role of central pro-inflammatory cytokines in the mediation of suppressory effect of LPS-treatment on the secretion of GnRH/LH is proven in the studies where the inhibition of synthesis of these mediators in the hypothalamus reduced the inhibition of the HPG axis activity [31, 32].

However, it was previously reported that LPS interacting TLR4 receptors are widespread in the neuroendocrine tissues including the hypothalamus and the AP [33–36]. TLRs are present in the brain, where their expression is believed to be limited to glial cells (microglia, astrocytes, and oligodendrocytes [37]. Thus, it cannot be excluded that LPS action on the activity of reproductive axis may also have direct character. In our previous study it was shown that LPS may directly affect the secretory activity of the pituitary gland. It has been found that endotoxin affects beta subunit of luteinizing hormone (*LH-β*) gene expression in the ex-vivo AP explants [38]. However, there is some evidence that LPS may also influence the HPG activity acting at the hypothalamic level. It was found that the influx of some quantities of endotoxin through the BBB occurs during inflammation. The measurable amount of iodine-radiolabelled LPS crossing the BBB and reaching the brain parenchyma was demonstrated in rats [39] and mice [40]. The latest results from Vargas-Caraveo et al. [41], suggest that LPS infiltrates in the brain in physiological conditions, possibly through a lipoprotein transport mechanism, and it is bound to its receptors in blood-brain interfaces. This fact suggests that the neuroinflammation caused by immune stress may be induced not only by pro-inflammatory cytokines crossing the BBB but also by the direct action of LPS reaching the CNS. It is known that neuroinflammatory processes may negatively influence the GnRH/LH secretion, therefore the central action of LPS could be involved in the suppression of HPG axis activity. It could be assumed that activation of the TLR4 signaling pathway in the hypothalamus plays an important role in this process.

The research hypothesis assumes that acting centrally bacterial endotoxin inhibits the GnRH/LH secretion in ewes and that blockade of TLR4 using CD14/TLR4 antagonist in the hypothalamus would reduce this suppressory action of LPS.

The aim of this study was to investigate the influence of intracerebroventricular (icv) injection of CD14/TLR4 antagonist on GnRH/LH secretion in anestrous ewes during central inflammation induced by icv administration of LPS.

## Methods

### Animals

The study was performed on 18 adult, 3-year-old Blackhead ewes in the anestrous season (April-May). All animals were in good condition (body condition score of 3 in a 5-point scale). The animals were maintained indoors in individual pens and exposed to natural daylight. The ewes were well adapted to the experimental conditions; always had visual contact with their neighbors even during the experimental period, to prevent the stress of social isolation. The animals were fed a constant diet of commercial concentrates with hay and water available ad libitum. One month before the start of the experiment all groups of ewes were cannulated with stainless steel guide cannulas (1.2 mm o.d.) into the third ventricle under stereotaxic control [42]. The guide cannula was fixed to the skull with stainless steel screws and dental cement. The correct placement of the guide cannula into the third ventricle was established by the efflux of the CSF from cannula during the surgery. Additionally, the placement of the cannula was checked by inspection of the brain after decapitation. All animals had a venous catheters implanted into jugular vein the day before the experiment. All procedures were performed with the consent of the Local Ethics Committee of Warsaw University of Life Sciences – SGGW, resolution No. 22/2013 of 22 May 2013.

### Experimental procedures

The animals (*n* = 18) were randomly assigned to three experimental groups: (1) control group (*n* = 6) that received icv injection of vehicle solution (150 μL/animal) containing 143 μL Ringer Locke solution (RLs) and 7 μL dimethyl sulfoxide (DMSO)/ethanol (1 vol: 1 vol) into the third ventricle of the brain; (2) LPS group (*n* = 6) that received icv injection of LPS from *E.coli* 055:B5 (Sigma Aldrich, St. Louis, MO, USA) solution (250 μg LPS dissolved in 150 μL vehicle solution/animal); (3) anti-TLR4 group (n = 6) that received icv injection of CD14/TLR4 antagonist IAXO-101 (Innaxon, Tewkesbury, UK) solution (150 μL/animal) consisting of 143 μL RLs and 7 μL 2 mmol/L stock solution of IAXO-101 (5 mg IAXO-101 dissolved in 3,085 μL DMSO/ethanol (1 vol: 1 vol) and 30 min later LPS solution (250 μg LPS dissolved in 150 μL vehicle solution/animal). IAXO-101 is a synthetic CD14/TLR4 antagonist modulating TLR4 activities in vitro, described to interfere with human, rat and mouse TLR4/CD14 signaling. The efficiency of the LPS treatment to induce an inflammatory response in the animal was estimated basing on the measurement of the body temperature. In all LPS treated animals the increase of body temperaturę was observed, while in CD14/TLR4 treated animals there was a complete suppression of LPS-induced fever (data not shown).Jugular blood samples from each ewe were taken for LH and cortisol measurement at 15 min intervals, beginning 2 h before and continuing 3 h after icv administration of reagents or control vehicle. The animals were euthanized immediately after the experiment (3 h after icv administration of reagents or vehicle), the brains were rapidly removed from the skulls and then chosen hypothalamic structures such as preoptic area (POA), anterior hypothalamus (AHA), medial basal hypothalamus (MBH) and median eminence (ME) and APs were dissected. All tissues were frozen immediately after collection in liquid nitrogen and were stored in − 80 °C until assay.

### Assays

#### Radioimmunoassay for plasma hormones (LH and cortisol)

The concentration of LH in plasma was assayed by the radioimmunoassay (RIA) double antibody method using anti-ovine-LH and anti-rabbit-γ-globulin antisera and ovine standard (teri.oLH; Tucker Endocrine Research Institute, Atlanta, GA, USA) as described by Stupnicki and Madej [43]. The sensitivity was 0.3 ng/mL; intra- and interassay coefficients of variation were 8.3% and 12.5%, respectively.

The cortisol concentrations were determined by the RIA method according to Kokot and Stupnicki [44], using rabbit anticortisol antisera (R/75) and HPLC grade cortisol standard (Sigma-Aldrich, Saint Louis, MI, USA). The assay sensitivity was 0.95 ng/mL and the intra- and interassay coefficients of variation were 10% and 12%, respectively.

#### Relative gene expression assays

Total RNA from hypothalamic and pituitary tissues was isolated using NucleoSpin RNA II Kit (MACHEREY-NAGEL Gmbh & Co Düren, Germany) according to manufacturer’s instructions. The purity and concentration of isolated RNA were quantified spectrophotometrically by measuring the optical density at 260 and 280 nm in the NanoDrop 1000 instrument (Thermo Fisher Scientific Inc., Waltham, USA). The RNA integrity was verified by electrophoresis using 1% agarose gel stained with ethidium bromide. Maxima First Strand cDNA synthesis Kit for RT-qPCR (Thermo Fisher Scientific Inc., Waltham, USA) was used to prepare cDNA synthesis. As a starting material for this PCR synthesis 2 μg of total RNA was used.

Real-time RT-PCR was performed using HOT FIREPol EvaGreen® qPCR Mix Plus (Solis BioDyne, Tartu, Estonia) and HPLC-grade oligonucleotide primers (Genomed, Warsaw, Poland). The primer sequences were designed using Primer 3 software (Table 1). One reaction mixture (in total 20 μL) contained: 4 μL of PCR Master Mix (5×), 14 μL of RNase-free water, 1 μL of primers (0.5 μL each primer, working concentration 0.25 μmol) and 1 μL of the cDNA template. The reactions were run on the Rotor-Gene 6000 instrument (Qiagen, Düsseldorf, Germany) with the following protocol was used: 95 °C for 15 min and 30 cycles of 95 °C for 10 s for denaturation, 60 °C for 20 s for annealing, and 72 °C for 10 s for extension. After the cycles, a final melting curve analysis under continuous fluorescence measurements was performed to confirm the specificity of the amplification.

**Table 1:** Specific primers used in real-time PCR for determining the expression of housekeeping genes and genes of interests

Gene	GenBank Acc. No.	Amplicon Size, bp	Sequence (5′ → 3′)	
			Forward	Reverse
*GAPDH* ^*a*^	NM_001034034	134	AGAAGGCTGGGGCTCACT	GGCATTGCTGACAATCTTGA
*ACTB* ^*b*^	U39357	168	CTTCCTTCCTGGGCATGG	GGGCAGTGATCTCTTTCTGC
*PPIC* ^*c*^	NM_001076910	131	ACGGCCAAGGTCTTCTTTG	TATCCTTTCTCTCCCGTTGC
*GnRH* ^*d*^	U02517	123	GCCCTGGAGGAAAGAGAAAT	GAGGAGAATGGGACTGGTGA
*GnRH-R* ^*e*^	NM_001009397	150	TCTTTGCTGGACCACAGTTAT	GGCAGCTGAAGGTGAAAAAG
*TLR4* ^*fg*^	AY957615	117	GGTTCCCAGAACTGCAAGTG	GGATAGGGTTTCCCGTCAGT
*IL-1β* ^*h*^	X54796.1	137	CAGCCGTGCAGTCAGTAAAA	GAAGCTCATGCAGAACACCA

References: ^a^[28]; ^b^[28]; ^c^[28]; ^d^[73]; ^e^[73]; ^f^[34]; ^g^[74]; ^h^[28]

Relative gene expression was calculated using the comparative quantification option of the RotorGene 6000 software 1.7 (Qiagen, Düsseldorf, Germany). Three housekeeping genes: glyceraldehyde-3-phosphate dehydrogenase (*GAPDH*), β-actin (*ACTB*), and cyclophilin C (*PPIC*) were tested. The BestKeeper was used to determine the most stable housekeeping gene for normalizing genes of interest expression. The BestKeeper was based on the pair-wise correlation analysis of all pairs of candidate genes [45] and calculated variations of all reference genes (SD ± Ct). *GAPDH* was chosen as the best endogenous control gene. It was characterized by the lowest SD ± Ct value and a good correlation coefficient with the remaining analyzed housekeeping genes. The results are presented as relative gene expression of the target gene versus housekeeping gene, and relative expression value and mean ± SEM. The average relative quantity of gene expression in control groups was set to 1.0.

#### ELISA assay for the GnRH concentration in the ME

Concentrations of GnRH in the ME were determined with a commercial GnRH ELISA kit dedicated for sheep (CUSABIO BIOTECH Co., Ltd., China). All steps in the assays were performed according to the manufacturer’s instructions as described by Herman et al. [32]. Shortly, the ME tissues were homogenized in 400 µL of phosphate buffered saline (0.02 mol). Then homogenates were subjected to two freeze-thaw cycles to further break the cell membranes. After that, the homogenates were centrifuged for 15 min at 1,500 × *g* in 4 °C. The incubation of plates and absorbance measurement at 450 nm were performed using a VersaMax reader (Molecular Devices, Sunnyvale, CA, USA). The assay sensitivity was 1.0 pg/mL. GnRH concentrations were normalized against the total protein content in each sample, assayed using the Bradford method [46].

### Statistical data analysis

All data are expressed as mean ± SEM. The results of blood hormones concentration obtained only after treatment period (from +1 h to +3 h after icv administration), GnRH content in the ME and all examined genes expression were subjected to a one-way ANOVA analysis. Before ANOVA was conducted its two assumptions were checked: normality (Shapiro-Wilk’s test) and homogeneity of the variances (Levene’s test). When a existence of interaction between factors was observed, a post hoc analysis using Tukey’s test was conducted to identify treatment effects. Statistical significance was defined as *P* < 0.05.

The statistical analysis was performed using STATISTICA software (Stat-Soft, Inc., Tulsa, OK, USA).

## Results

### Effect of icv administration of CD14/TLR4 antagonist on plasma hormones release during central inflammation induced by icv administration of LPS

**Fig. 1:**
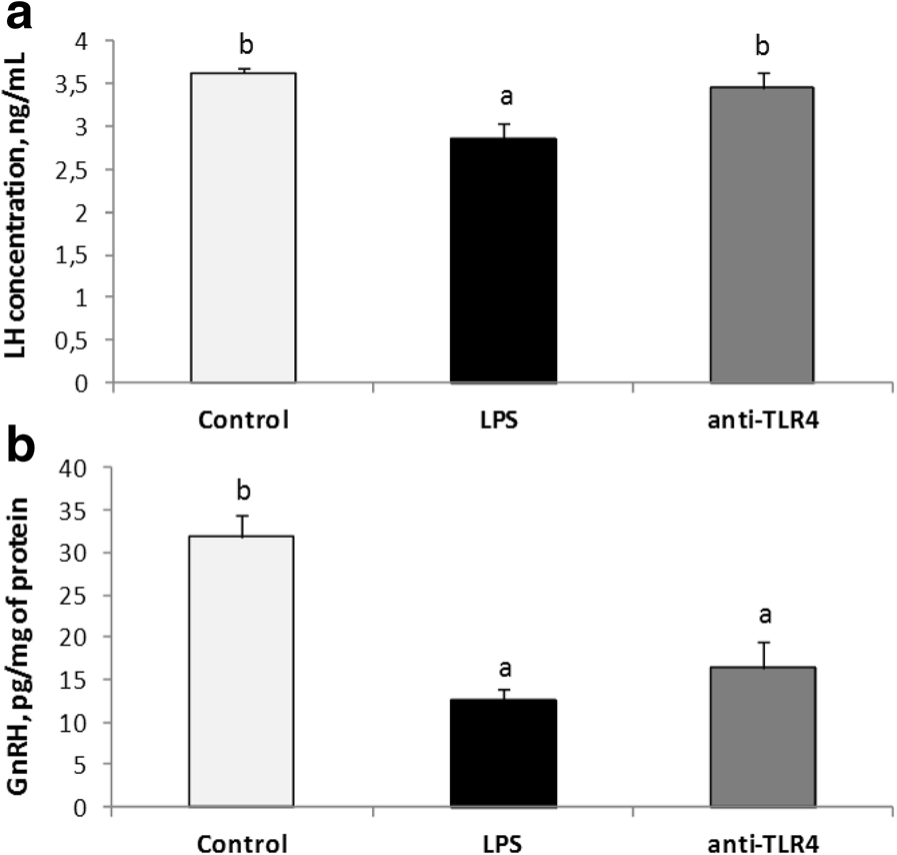
Mean (± SEM) concentration of plasma LH (panel A) and GnRH in the ME (panel B) after icv administration of CD14/TLR4 antagonist during central inflammation induced by icv administration of LPS. Different lowercase letters (**a**, **b**) indicate significant differences between groups at *P* < 0.05 (one-way ANOVA followed by Tukey’s post hoc test)

**Fig. 2:**
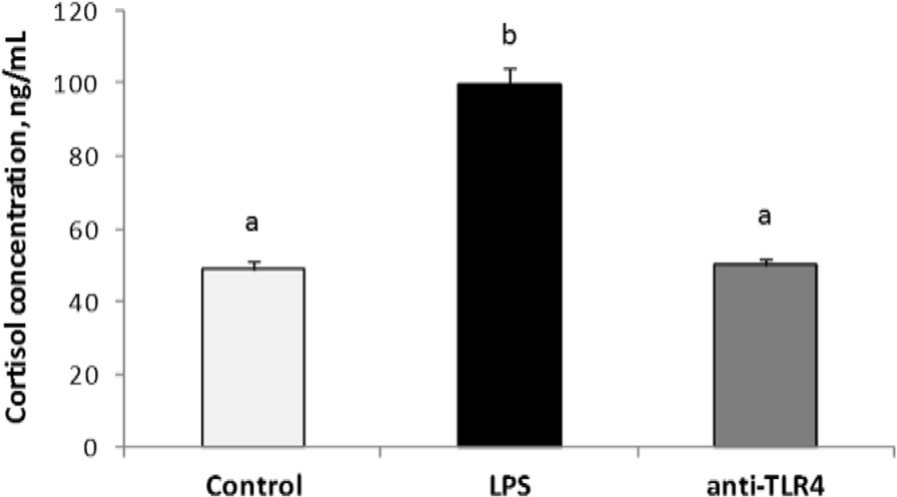
Mean (± SEM) concentration of plasma cortisol after icv administration of CD14/TLR4 antagonist during central inflammation induced by icv administration of LPS. Different lowercase letters (**a**, **b**) indicate significant differences between groups at P < 0.05 (one-way ANOVA followed by Tukey’s post hoc test)

Centrally administered LPS decreased (*P* < 0.05) plasma LH concentration in comparison with control group. Treatment with CD14/TLR4 antagonist reduced the inhibitory effect of LPS on LH release (Fig. 1a). LPS administration (icv) significantly (*P* < 0.05) increased plasma cortisol level and treatment with CD14/TLR4 antagonist prevented the influence of LPS (Fig. 2).

### Effect of icv administration of CD14/TLR4 antagonist on GnRH synthesis in the ME during central inflammation induced by icv administration of LPS

Centrally administered LPS decreased (*P* < 0.05) GnRH concentration in the ME as compared with control group. Treatment with CD14/TLR4 antagonist did not prevent the inhibitory effect of LPS on GnRH level (Fig. 1b).

### Effect of icv administration of LPS and CD14/TLR4 antagonist on *IL-1β* gene expression in the hypothalamus

**Fig. 3:**
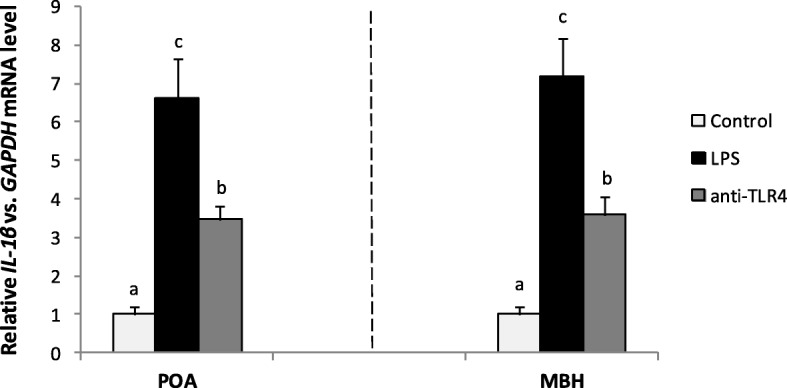
Mean (± SEM) relative IL-1 β mRNA in hypothalamic structures POA and MBH after icv administration of CD14/TLR4 antagonist during central inflammation induced by icv administration of LPS. Different lowercase letters (**a**, **b**, **c**) indicate significant differences between groups at *P* < 0.05 (one-way ANOVA followed by Tukey’s post hoc test)

Centrally administered LPS significantly increased (*P* < 0.05) *IL-1β* gene expression in the POA and in the MBH as compared with the control group. The central administration of CD14/TLR4 antagonist significantly (*P* < 0.05) reduced LPS dependent increase of *GnRH* gene expression in comparison with LPS group in these structures (Fig. 3).

### Effect of icv administration of CD14/TLR4 antagonist on *GnRH* and *GnRH-R* genes expression during central inflammation induced by icv administration of LPS

**Fig. 4:**
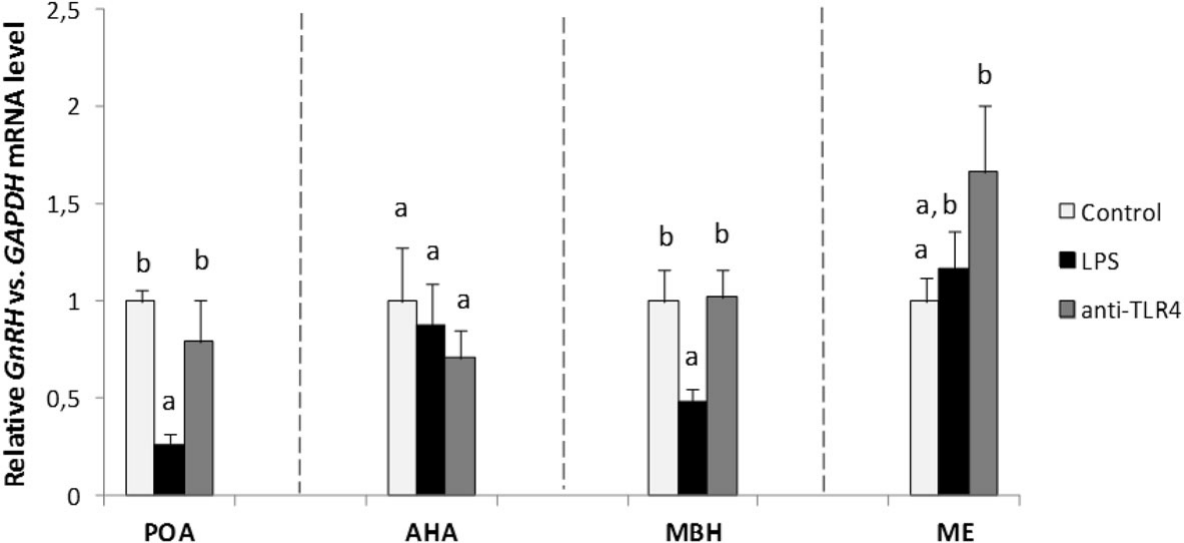
Mean (± SEM) relative *GnRH* mRNA expression in four hypothalamic structures (POA, AHA, MBH, ME) after icv administration of CD14/TLR4 antagonist during central inflammation induced by icv administration of LPS. Different lowercase letters (**a**, **b**) indicate significant differences between groups at *P* < 0.05 (one-way ANOVA followed by Tukey’s post hoc test)

*GnRH* and *GnRH-R* genes expressions were determined in four hypothalamic structures: POA, AHA, MBH and the ME. Icv injection of LPS significantly (*P* < 0.05) decreased *GnRH* gene expression in the POA and in the MBH as compared with the control group. The central administration of CD14/TLR4 antagonist significantly (*P* < 0.05) reduced LPS dependent suppression of *GnRH* gene expression in comparison with LPS group in these structures. In the ME gene expression of *GnRH* was significantly increased (*P* < 0.05) in anti-TLR4 group versus the control group (Fig. 4).

**Fig. 5:**
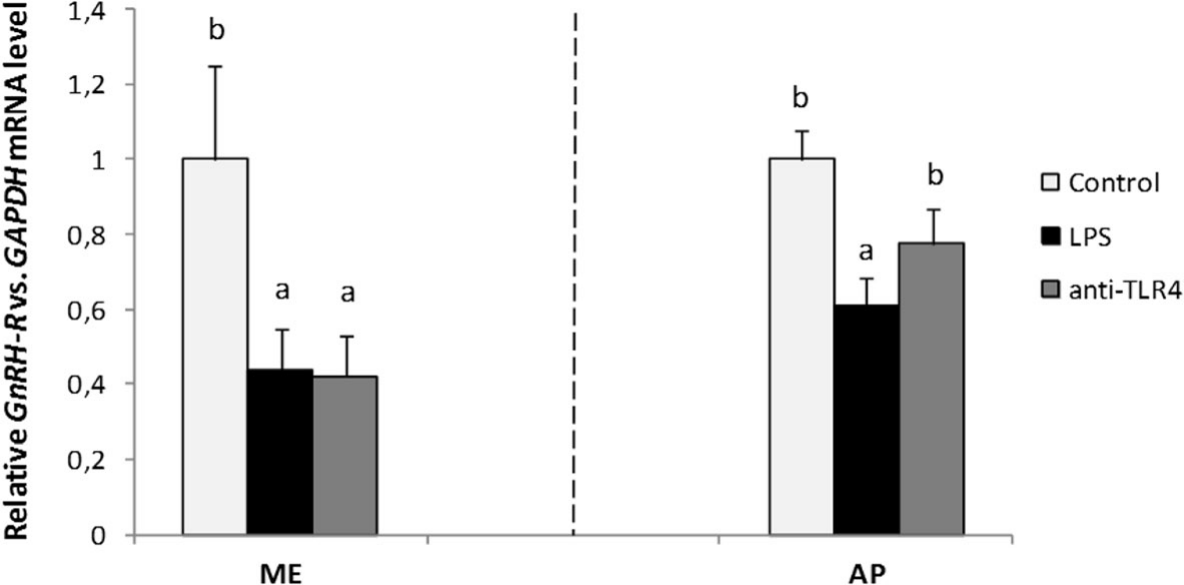
Mean (± SEM) relative *GnRH-R* mRNA expression in the ME and in the AP after icv administration of CD14/TLR4 antagonist during central inflammation induced by icv administration of LPS. Different lowercase letters (**a**, **b**) indicate significant differences between groups at *P* < 0.05 (one-way ANOVA followed by Tukey’s post hoc test)

Gene expression of receptor for GnRH was determined only in the ME and in the AP, because the amount of *GnRH-R* mRNA determined in the other examined hypothalamic structures (POA, AHA, MBH) was too low and so the quantitative analysis in these structures was enabled to be performed. Central injection of LPS significantly (*P* < 0.05) decreased *GnRH-R* mRNA level in the ME and in the AP in comparison with the control group. The central administration of CD14/TLR4 antagonist significantly (*P* < 0.05) reduced LPS dependent suppression of *GnRH-R* gene expression only in the AP (Fig. 5).

### Effect of icv administration of CD14/TLR4 antagonist on *TLR4* gene expression during central inflammation induced by icv administration of LPS

**Fig. 6:**
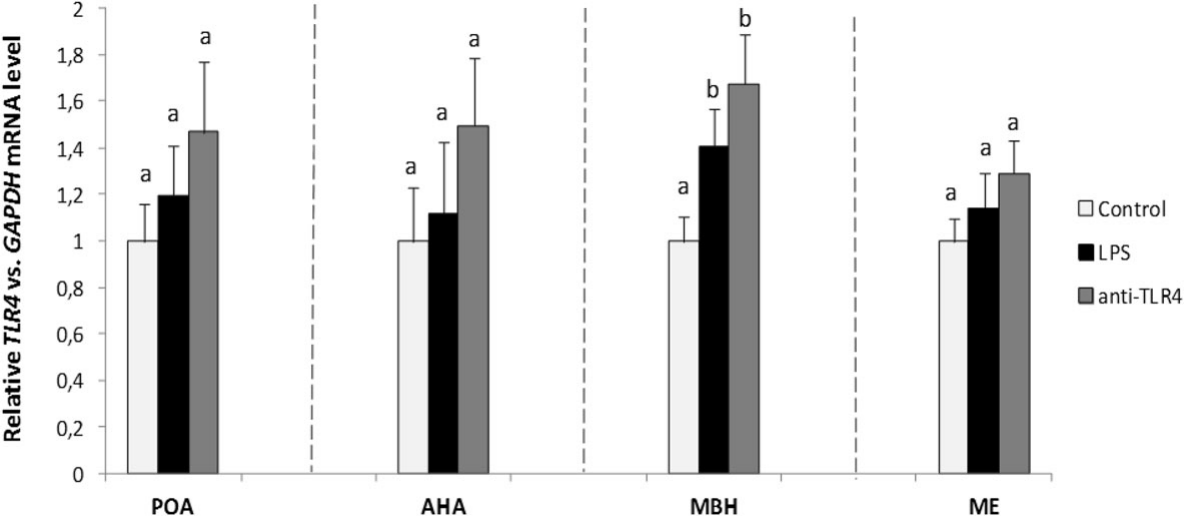
Mean (± SEM) relative *TLR4* mRNA expression in four hypothalamic structures (POA, AHA, MBH, ME) after icv administration of CD14/TLR4 antagonist during central inflammation induced by icv administration of LPS. Different lowercase letters (**a**, **b**) indicate significant differences between groups at *P* < 0.05 (one-way ANOVA followed by Tukey’s post hoc test)

*TLR4* gene expression was detected in four analyzed hypothalamic structures POA, AHA, MBH and the ME. Central injection of LPS and CD14/TLR4 antagonist with LPS increased (*P* < 0.05) *TLR4* mRNA level in the MBH as compared with the control group (Fig. 6).

## Discussion

Our study demonstrated that icv LPS administration elevated local synthesis of IL-1β in the hypothalamus of anestrous sheep and central administration of CD14/TLR4 antagonist reduces this stimulatory effect of LPS. The increased * IL-1β* gene expression testify the existence of inflammatory process in the hypothalamus. The stimulation of this cytokine gene expression in the hypothalamus of anestrous ewes was also described during peripheral inflammatory challenge triggered by intra venous LPS injection [27].

In our study it was demonstrated that centrally administered bacterial endotoxin could inhibit the secretion of GnRH directly at the hypothalamic level acting primary through its TLR4. It was found that icv administration of LPS reduced the level of *GnRH* mRNA in the hypothalamic structures such as the POA and MBH. However, the blockade of the TLR4 in the hypothalamus by administering its antagonist prior to icv injection of LPS, prevented lowering of the *GnRH* gene expression in these structures but not in ME.

Moreover, it was determined that centrally acting LPS reduced the accumulation of GnRH peptide in the ME, where most (in sheep approximately 70%) of GnRH-ergic neuron terminals are located [47]. Neuropeptide GnRH is generally synthesized in the neuronal cells bodies located mainly in the POA and in a certain amount also in other hypothalamic structures such as MBH, and after synthesis is transported to the nerves terminals in the ME and there is stored till release. It is released into the capillaries of the hypothalamic-pituitary portal system in a pulsatile manner. This decapeptide is transported with blood to the gonadotrophes in the AP and regulates secretion of gonadotropins [48]. It has been shown that increased synthesis and accumulation of GnRH in the ME leads to increased pulse frequency, which is a main factor leading to pre-ovulatory discharge of gonadotropins, and therefore an important element of positive feedback [49]. Therefore, LPS-dependent reduction of the GnRH accumulation in the ME may exert a profound negative effect on this peptide release and downstream LH secretion. However, mRNA encoding GnRH seems to be not synthesized directly in the ME but it is speculated that this mRNA is transported to the ME from the other hypothalamic structures. The physiological roles of this mRNA as well as the mechanisms regulating its transport to the ME are not well understood. It is considered that this local *GnRH* mRNA may support the secretion of GnRH from the nerves terminals but this issue has not been proved yet. Therefore the amount of GnRH peptide does not have to reflect the changes in the level of *GnRH* mRNA in this structure. This may be the reason for the lack of parallelism the results concerning changes in mRNA *GnRH* and concentration of GnRH peptide in ME in anti TLR4 group. The present results showing that blockade of TLR4 at the level of hypothalamus may reduce some negative effects of centrally acting endotoxin on the GnRH secretion are generally consistent with the results of our previous study on anestrous ewes with endotoxin-induced inflammation. In that study it was found that blocking of TLR4 components in the hypothalamus, as well as central administration of anti-LPS antibody to the third ventricle of the brain, reduced some suppressive effect of inflammation on the secretion of GnRH/LH [35]. Thus, it can be concluded that blocking TLR4 at the hypothalamic level led to unblocking the inhibition of GnRH secretion, which may confirm the direct action of endotoxin in the hypothalamus in the inhibition of reproductive axis in anestrous ewes. There is no data concerning the influence of peripherally administered TLR4 antagonists on the disturbed by immune challenge GnRH/LH secretion at the central level. However, the study on rats proved that the peripheral administration of TLR4 selective inhibitor TAK-242 prevents the neuroinflammation [50].

In the present study it was also determined that centrally administered LPS decreased *GnRH-R* gene expression in the ME and AP. Additionally, the blockade of TLR4 reduced the LPS-dependent decrease in the gene expression of *GnRH-R* in the AP. It seems that reduced *GnRH-R* gene expression in the ME after icv injection of LPS may result from decreased feedback stimulation of GnRH neurons by GnRH. This phenomenon may be caused by lowered release of this peptide into the portal system by nerve terminals located in the ME [51–53]. Regulation of *GnRH-R* gene expression in AP is influenced by a multitude of factors including gonadal steroid hormones, inhibin, activin, and perhaps the most importantly, GnRH itself. Normal secretory patterns of GnRH appear to be essential for maintaining a full complement of GnRH-R in the anterior pituitary gland [53]. Decreased *GnRH-R* gene expression after LPS injection in the AP observed in our experiment may result from reduced GnRH secretion. It can be assumed that lowered *GnRH-R* gene expression led to lowered GnRH-R expression on the pituitary cells and reduced sensitivity of these cells to GnRH stimulation. Down-regulating *GnRH-R* gene expression is probably one of the mechanisms by which LPS influences LH secretion at the AP level. Our results are consisted with the previous studies carried out on anestrous ewes where systemically injected endotoxin lowered *GnRH-R* gene expression in the pituitary gland [54].

Parallel to the reduced GnRH secretion a decreased LH release after icv LPS administration which was reduced by TLR4 antagonist was observed. A lower GnRH secretion could lead to decreasing the GnRH-R expression and as a consequence to the reduction of LH secretion. The dose-dependent inhibitory effect of ventricularly administered LPS on LH secretion have been successfully shown in the experiment on rats, where two doses of endotoxin (5 and 25 μg) induced a significant decrease in plasma LH level [55]. It was also found that administration of anti-IL-1α rabbit serum and recombinant human IL-1 receptor antagonist significantly attenuated the LPS-induced reduction of plasma LH levels but only in the early period after the administration, with no influence on the late period. These results may indicate that IL-1 could participate in the reduction of plasma LH levels induced by icv- injected LPS, especially during the first 60 min, and that the later effect of LPS depends on factors other than IL-1 [55].

In the current study *TLR4* gene expression was determined in selected hypothalamic structures (POA, AHA, MBH, ME) which suggests that the activity of this region may be influenced by ligands of this receptor. This finding is in line with the results of our previous studies in which expression of *TLR4* mRNA in the ovine hypothalamus was found [34, 35]. Moreover, it was demonstrated that peripheral inflammation induced by intravenous administration of endotoxin resulted in increased *TLR4* gene expression [34]. In the study conducted on rats where cellular distribution of TLR4 in the hypothalamus was evaluated by double-immunofluorescence staining of hypothalamic sections, it was shown that the most TLR4-positive cells were microglia cells [56]. In the present study it was shown that icv administration of LPS increased *TLR4* gene expression in the MBH. The presence of TLR4 in the hypothalamus enable central action of endotoxin and its influence on the neuroendocrine system. However, this action may have an indirect character and involve mediators synthesized as a result of transduction of these receptor signal. Pro-inflammatory cytokines which can mediate the inhibitory effect of icv-administered LPS at the level of the hypothalamus [3] seem to play an important role in this indirect action [13, 18, 19]. Kaisho and Akira [57] have demonstrated that TLR4 signal transduction induced pro-inflammatory cytokine synthesis. Feng et al. [58] have shown that an inflammatory response in the brain induced by LPS leads to the release of IL-1β into the CSF by endothelial cells, and, consequently to the secretion of cytokines such as IL-1β, IL-6 and TNFα. Moreover, in the studies on ewes it was shown that these pro-inflammatory cytokines are synthesized directly in the hypothalamic area involved in the GnRH-ergic activity [27, 28, 59]. Centrally acting mediators are considered to be responsible for anti-reproductive effect of LPS, in particular IL-1β and TNFα, which are involved in suppression of GnRH/LH secretion [3, 60, 61]. Particularly, IL-1β plays a crucial role in inhibiting the HPG axis activity at hypothalamic level, as it was confirmed in rats [29] and sheep [30].

However, disturbances found in the GnRH secretion after central LPS treatment at least partially may result from the activation of the HPA axis activity. The study demonstrated that icv administration of LPS caused a significant increase in blood plasma level of cortisol, commonly regarded as a marker of stress response. The increase in plasma level of this hormone proves the activation of the HPA stress axis, which can suppress the activity of the HPG axis [62–64]. It has been documented that stress causes changes in the activity of the HPG axis, and the nature of these changes depends on the type of stressor, duration of exposure to that stressor and physiological state of the animal [65, 66]. Hormones and peptides released during stress response play an important role in modulating the influence of stress on the reproductive functions of the organism. The effect of these factors can occur at all levels of the HPG axis [67]. It was shown that immune stress stimulated secretion of corticotropin-releasing hormone (CRH) from the hypothalamus, adrenocorticotropic hormone (ACTH) from the pituitary gland as well as cortisol, corticosterone/cortisone from the adrenal cortex [68, 69]. All mentioned factors have a suppressive effect on the HPG axis [63, 64]. Among others, it has been shown that elevated cortisol level in peripheral blood inhibit LH secretion by decreasing the sensitivity of gonadotropic cells to GnRH [62, 70, 71]. Generally, it is known that stress has a regulatory effect on *GnRH* gene expression in the ovine hypothalamus [66, 72].

## Conclusions

The results of present in vivo experiment indicate that blockade of TLR4 reduces the inhibitory effect of centrally acting LPS on the *GnRH* gene expression and LH release in anestrous ewes. This suggests that TLR4 and its ligand could be involved in the induction of sickness behavior already in the hypothalamus. Some negative effects of bacterial infection on the HPG axis activity at the hypothalamic level may be caused by LPS crossing the BBB. However, the effect of bacterial endotoxin can also be mediated by the activation of pro-inflammatory cytokines, so it is necessary to undertake further research on the mechanism of LPS action in the organism. Identification of the central pathways responsible for the transmission of LPS signal to GnRH neurons and the role of TLR4 in this process is essential to understand the immune stress-induced reproductive disorders in humans and animals. This knowledge will be helpful in developing newer and more effective treatments for such dysfunctions. Manipulation of TLR4 - mediated immune responses could be a potential approach to treat a variety of inflammation diseases at the CNS level.

## Abbreviations

ACTB: β-actin
AHA: Anterior hypothalamus
AP: Anterior pituitary gland
BBB: Blood-brain barrier
CD: Cluster of differentiation
CNS: Central nervous system
CP: Choroid plexus
CSF: Cerebrospinal fluid
DMSO: Dimethyl sulfoxide
GAPDH: Glyceraldehyde-3-phosphate dehydrogenase
GnRH: Gonadotropin-releasing hormone
GnRH-R: Receptor for GnRH
HPA: Hypothalamic-pituitary-adrenal
HPG: Hypothalamic-pituitary-gonadal
Icv: Intracerebroventricular
IL: Interleukin
LBP: LPS-binding protein
LH: Luteinizing hormone
LH-β: Beta subunit of luteinizing hormone
LPS: Lipopolysacccharide
MBH: Medial basal hypothalamus
MD-2: Myeloid differentiation factor 2
ME: Median eminence
POA: Preoptic area
PPIC: Cyclophilin C
RIA: Radioimmunoassay
TLR: Toll-like receptor
TNF: Tumor necrosis factor
